# Role of perineuronal nets in Alzheimer's Disease‐associated social memory impairment

**DOI:** 10.1002/alz70855_103261

**Published:** 2025-12-23

**Authors:** Lata Chaunsali

**Affiliations:** ^1^ University of Virginia, Charlottesville, VA, USA

## Abstract

**Background:**

Alzheimer's disease (AD) is a progressive neurodegenerative disorder affecting approximately 55 million people worldwide. Key pathological features of AD include the accumulation of beta‐amyloid plaques and hyperphosphorylated tau tangles. However, recent studies have also identified changes in the extracellular matrix (ECM) within the brains of both humans and mouse models of AD. In several brain regions, the ECM forms a specialized, lattice‐like structure known as perineuronal nets (PNNs). These nets predominantly surround PV‐expressing GABAergic neurons but are also found around excitatory neurons in the hippocampal CA2 region. Emerging evidence has linked PNN alterations to various neurological disorders, including AD. Given that PNNs play a critical role in learning and memory, we hypothesized that cognitive decline and memory deficits in AD might result from altered PNNs.

**Method:**

Using the 5XFAD mouse model of AD, we explored the role of PNNs in memory dysfunction through immunohistochemistry, qPCR, behavioral tests, gene knockout experiments, and pharmacological interventions.

**Result:**

We discovered that PNNs in the hippocampal CA2 region are disrupted at an unexpectedly early stage in the 5XFAD model of AD. Mice lacking CA2 PNNs exhibited impaired social memory. Investigating the underlying mechanisms, we observed an upregulation of ECM remodeling enzymes, including several matrix metalloproteinases (MMPs). To establish a causal link between PNN disruption and social memory impairment, we experimentally disrupted CA2 PNNs in wild‐type mice. These mice displayed social memory deficits similar to those observed in AD mice. Notably, treatment with a broad‐spectrum MMP inhibitor, GM6001, prevented PNN disruption and preserved social memory in 5XFAD mice.

**Conclusion:**

Our findings suggest that disrupted PNNs in the hippocampal CA2 region contribute to social memory dysfunction in the 5XFAD mouse model of AD. Targeting ECM remodeling enzymes to prevent PNN disruption may offer a promising therapeutic strategy for addressing memory deficits associated with AD.

